# Transverse myelitis as a presenting feature of late onset systemic lupus erythematosus

**DOI:** 10.4103/0256-4947.51795

**Published:** 2009

**Authors:** Hani Almoallim, Majidah Bukhari, Leena Alwafi, Gassan Wali

**Affiliations:** King Faisal Specialist Hospital and Research Center, Jeddah, Saudi Arabia

**To the Editor:**

**Figure 1:**
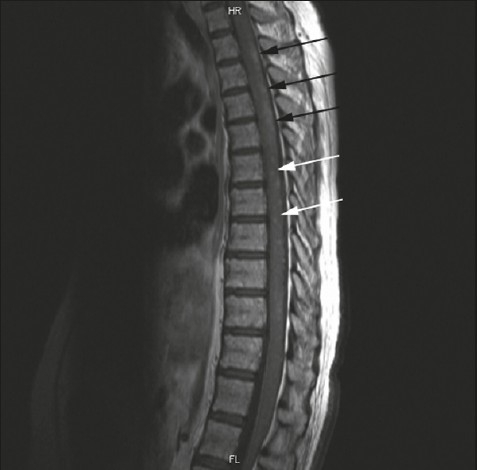
MRI of the thoracic spine (sagittal view T1-weighted post-gadolinium image) showing some multifocal areas of enhancement within the cord that predominantly involve the posterior aspect of the cord (black arrows).There are also extensive focal small enhancing lesions (white arrows).

Late-onset systemic lupus erythematosus (SLE) is the type of SLE whose manifestations reportedly begin after the age of 50^1^–^3^ or 65 years.^4^ The prevalence of transverse myelitis (TM) in SLE patients is 1% to 2%.^5^ It can occur as the initial manifestation of SLE in up to 39% of patients.^5^ TM as a presenting feature of late-onset SLE is rare. Only a few cases have been reported.^5^–^7^ We report on a previously healthy 65-year-old female patient who presented to our hospital with a month-long history of progressive weakness, numbness of the lower limbs and unsteady gait that was associated with loss of bowel and bladder control. She also reported an intermittent history of fever but no weight loss, or night sweats, headache, vomiting or seizure activity. She was conscious, alert and oriented to place, person and time. Her temperature was 38.0°C, heart rate 123 beats/minute, blood pressure 155/80 mm Hg and respiratory rate 18 per minute with 98% oxygen saturation. Examination of the cranial nerves was unremarkable. Motor examination showed hypotonia in the lower limbs with a power of 0/5, absent reflexes and downgoing planter responses bilaterally. There was loss of all sensation up to the nipples at the level of T4. Investigations at presentation showed neutrophilic leukocytosis with lymphopenia, an elevated PTT of 45 seconds^1^ and an erythrocyte sedimentation rate of 5 seconds with normal renal and hepatic profiles. Cerebrospinal fluid (CSF) analysis showed pleocytosis; white blood cells (WBC) of 3.71×10^9^/L with 75% lymphocytes, glucose of 5.5 mmol/L, protein of 1419 mg/L and negative cultures for all bacteria and viruses including acid fast bacilli. PCR for acid fast bacilli was negative as well. Urine culture was positive for *E. coli.* MRI of the spine and brain with T1-and T2-weighted sagittal and axial images including a magnetic resonance angiogram showed normal brain parenchyma and diffuse hyperintensity within the cord at the level of thoracic spine T1 extending up to T10. The diagnosis of idiopathic TM was made. She was treated with pulse therapy with IV methylprednisolone (1 gram) for 5 days and intravenous ceftriaxone. Her condition improved significantly with power increased from 0/5 to 3/5 in the lower limbs. Two weeks later, despite taking an oral steroid, power over her lower limbs was 0/5 again. The repeat MRI showed multifocal areas of significant hyperintensity within the thoracic spine with multifocal enhancement in the posterior aspect of the cord post gadolinium (Figure 1). Anti-tuberculosis medications were initiated and discontinued when acid fast bacilli were not isolated by cultures and PCR technique. Rheumatological assessment after approximately 1 month of hospital admission revealed a positive antinuclear antibody (ANA) test with a titer of 1:320. The antids DNA antibodies were repeatedly positive at 32.9 IU/mL (normal less than 10 IU/mL). Lupus anticoagulant was positive as well. She was diagnosed with TM as a presenting manifestation of SLE with antiphospholipid antibodies. Another pulse therapy with IV methylprednisolone (1 gram) for 5 days was started. The patient preferred not to receive any additional therapeutic interventions. Her weakness improved significantly with a power of almost 5/5 over limb girdle muscles 8 months after discharge. The repeat MRI demonstrated some improvement in previously present hyperintensity within the upper thoracic cord.

The management of TM continues to represent a major therapeutic challenge for clinicians in daily practice. The ideal drugs, doses, and the length of treatment are not yet well defined. In older studies, most patients were treated with IV corticosteroids alone, whereas more recently some centers prefer a more aggressive approach with IV methylprednisolone pulse therapy plus IV cyclophosphamide. There were no clear differences between these drugs in five cases of TM when both were studied against each other after induction therapy with methylprednisolone.^8^ However, several studies reported good to fair functional outcomes with combined treatment.^6^ The majority of TM cases reported in the literature were positive for antiphospholipid antibodies: 73% in one series^6^ and 55% to 64% in another.^5^ One of the strongest risk factors for the development of significant neuropsychiatric damage was the presence of antiphospholipid antibodies.^9^ This has resulted in the introduction of anticoagulant therapy in the management of TM patients with positive antiphospholipid antibodies, but this practice remains controversial. Plasmapheresis has been used to complement this treatment regimen,^5^,^10^,^11^ but it is still unclear if it has any additional therapeutic benefit. There are recent reports on the successful use of anti-CD20 in patients with TM.^12^,^13^ The patient described in this letter had significant improvement with the use of steroids alone. TM in the elderly might be controlled with the use of a large dose of steroid.
